# Hepatitis B, HIV, and Syphilis Seroprevalence in Pregnant Women and Blood Donors in Cameroon

**DOI:** 10.1155/2016/4359401

**Published:** 2016-08-08

**Authors:** Jodie Dionne-Odom, Rahel Mbah, Nicole J. Rembert, Samuel Tancho, Gregory E. Halle-Ekane, Comfort Enah, Thomas K. Welty, Pius M. Tih, Alan T. N. Tita

**Affiliations:** ^1^University of Alabama at Birmingham, Birmingham, AL 35294, USA; ^2^Cameroon Baptist Convention Health Services (CBCHS), P.O. Box 1, Nkwen, Bamenda, Cameroon; ^3^University of Buea, P.O. Box 12, Buea, Cameroon

## Abstract

*Objectives*. We estimated seroprevalence and correlates of selected infections in pregnant women and blood donors in a resource-limited setting.* Methods*. We performed a cross-sectional analysis of laboratory seroprevalence data from pregnant women and voluntary blood donors from facilities in Cameroon in 2014. Rapid tests were performed to detect hepatitis B surface antigen, syphilis treponemal antibodies, and HIV-1/2 antibodies. Blood donations were also tested for hepatitis C and malaria.* Results*. The seroprevalence rates and ranges among 7069 pregnant women were hepatitis B 4.4% (1.1–9.6%), HIV 6% (3.0–10.2%), and syphilis 1.7% (1.3–3.8%) with significant variability among the sites. Correlates of infection in pregnancy in adjusted regression models included urban residence for hepatitis B (aOR 2.9, CI 1.6–5.4) and HIV (aOR 3.5, CI 1.9–6.7). Blood donor seroprevalence rates and ranges were hepatitis B 6.8% (5.0–8.8%), HIV 2.2% (1.4–2.8%), syphilis 4% (3.3–4.5%), malaria 1.9%, and hepatitis C 1.7% (0.5–2.5%).* Conclusions*. Hepatitis B, HIV, and syphilis infections are common among pregnant women and blood donors in Cameroon with higher rates in urban areas. Future interventions to reduce vertical transmission should include universal screening for these infections early in pregnancy and provision of effective prevention tools including the birth dose of univalent hepatitis B vaccine.

## 1. Introduction

Many pregnant women in sub-Saharan Africa are infected with or exposed to pathogens that can be transmitted vertically. These include HIV, hepatitis B virus (HBV), hepatitis C virus (HCV), malaria, and syphilis. The usual mechanism for perinatal transmission of HBV, HCV, and HIV is exposure to infected maternal blood and fluids at the time of delivery. Vertical transmission of syphilis is transplacental and it occurs in 60–90% of pregnant women with early disseminated disease [1]. Pregnancy is also associated with an increased susceptibility to certain infections, including HIV and malaria [2–4].

Screening for infections during antenatal care is important since many infections are asymptomatic yet treatable. Screening practices are standardized in high-income countries, but access to antenatal care testing is more limited in low- and middle-income countries (LMIC) where infection rates are elevated [5]. Many women in LMIC also present later for antenatal care, leaving less time for intervention when infection is detected. In sub-Saharan Africa, access to screening during pregnancy has improved over the past decade, particularly as HIV prevention programs have been scaled up [6, 7]. Antenatal screening for HIV and syphilis is now commonly performed, but screening for hepatitis B is not routine. Since HBV remains an endemic infection in many countries, the World Health Organization (WHO) recommends the administration of birth dose of univalent hepatitis B vaccine to all newborns to help reduce vertical transmission and routine antenatal screening guidelines are being developed [8, 9]. Pregnant women in low-income countries also have a heightened risk of acquiring infection due to an unsafe blood supply. The prevalence of anemia in pregnancy is as high as 70% in sub-Saharan Africa and postpartum hemorrhage is the major cause of global maternal mortality [10, 11]. Blood transfusion during pregnancy and the peripartum period can be necessary, but it comes with a risk of transfusion-transmitted infection. Infection rates in the locally available blood supply may be elevated, but WHO recommended screening for bloodborne pathogens is not universally performed [12].

The Cameroon Health Initiative at the University of Alabama at Birmingham (CHI UAB) is a collaborative partnership with US and Cameroon-based investigators to improve maternal and infant health outcomes with infection as one major focus area. In order to obtain baseline information to guide future interventions, we sought to estimate the prevalence and correlates of HBV, HIV, HCV, malaria, and syphilis infection among pregnant women and blood donors at multiple program sites in Cameroon.

## 2. Methods

We performed a cross-sectional seroprevalence study using laboratory data collected from the antenatal clinic (ANC) (HBV, HIV, and syphilis testing) and blood donor registries (HBV, HIV, syphilis, HCV, and malaria testing) in 2014 at four large hospital facilities in geographically distinct areas of Cameroon.

### 2.1. Study Population and Setting

The Cameroon Baptist Convention Health Services (CBCHS) is a nongovernmental health care organization that has been active for more than 70 years with over 80 facilities in 6 of the 10 regions of the country. The study was implemented at 4 of the major facilities in 3 regions of Western Cameroon: Banso Baptist Hospital (Banso), Mbingo Baptist Hospital (Mbingo), Baptist Hospital Mutengene (Mutengene), and Mboppi Baptist Hospital in Douala (Mboppi). Pregnant women underwent routine screening for hepatitis B, syphilis, and HIV at their initial antenatal care visit or at the time of delivery. For HIV testing, an opt-out verbal consent has been standard practice since 2000 and screening acceptance rates now approach 100%. The cost of laboratory testing is included in the ANC registration fee which is approximately $15 and HIV testing is provided free of charge.

Blood donors from the same four facilities underwent verbal consent and routine clinical screening for hepatitis B, hepatitis C, syphilis, and HIV infections. Malaria screening was only performed at one facility site (Banso). Any positive screening test result led to disqualification of the donated blood sample. The system for blood donation relies on voluntary donations and donors are frequently relatives or friends of the recipient. Donors do not receive compensation and screening is performed free of charge. The cost of testing is transferred to the recipient of the blood and one unit of blood costs $30.

### 2.2. Clinical Tests

The hepatitis B testing used for pregnant women and blood donors was a hepatitis B surface antigen (HBsAg) immunoassay rapid test with a reported sensitivity of 98% and a 15-minute turnaround time (ABON, Alere) [13]. For HIV screening, the Determine HIV-1/2 rapid antibody test was used for both blood donors and antenatal patients (Abbott Laboratories, Chicago, Illinois, now Alere). It is a lateral flow assay with a 15-minute turnaround time. The Determine HIV-1/2 rapid test used has demonstrated 100% sensitivity and 99.8% specificity in pregnancy [14]. A second rapid HIV test (the First Response HIV Card Test) was performed on antenatal patients who had a positive Determine test. The sensitivity of the First Response HIV Card Test is 99.8% and the specificity is 100% [15, 16]. If the Determine test was positive but the First Response test was negative, repeat testing was recommended in one month using the First Response test. Positive HIV results for this analysis reflect those with two positive rapid antibody tests. All blood donors were tested with both HIV antibody tests. The HIV tests were chosen based on WHO recommendations, but there is no current WHO or CDC recommendation to use a particular antibody test for hepatitis B and hepatitis C screening.

A rapid test was also used for syphilis screening in pregnant women and blood donors. The Syphilis Ultrarapid Test Strip (ABON, Alere) has 20-minute turnaround time. It is a chromatographic treponemal antibody immunoassay and the sensitivity of similar treponemal tests has been documented at 96–100% compared to a gold standard of TPPA [17]. Nontreponemal testing was not performed to help distinguish active from prior syphilis infection.

Hepatitis C antibody testing was performed for blood donors (DFI Healthmate HCV Ab, South Korea). This test is a 3rd generation EIA with reported 98% sensitivity [18]. Malaria testing was performed on blood donation samples at one facility (Banso). The testing used was a rapid test called Care Start pLDH which detects* P. falciparum *(Pf) specific lactate dehydrogenase (Pf-pLDH) and an antigen common to all four plasmodium species (pan-pLDH). The sensitivity of this test is 94.7% for Pf compared to PCR testing although the sensitivity for non-Pf species is lower (74% for* P. vivax*, 32% for* P. malariae*, and 25% for* P. ovale*) [19]. Over 90% of the malaria in Cameroon is caused by* P. falciparum* [20].

### 2.3. Data Management and Statistical Analysis

The laboratory screening registers were reviewed and deidentified data were abstracted for analysis. Individual-level results for each infection were collected for analysis from three sites (Banso, Mbingo, and Mutengene), and aggregated data was available from the 4th site (Mboppi). In addition, age and location of residence (categorized into urban, suburban, and rural) were collected.

Statistical analysis was performed using SAS 9.3. The primary outcomes of interest were the prevalence of each of the infections in pregnant women and in blood donors. The Kruskal-Wallis test was used to compare the median age of pregnant women and blood donors at each site. The Chi square test was used to compare categorical variables. We estimated the prevalence and 95% CI of each infection in women and compared the prevalence across facilities. Individual-level patient data was merged from the three sites where it was available and demographics were examined as potential factors associated with infection prevalence. Logistic regression modeling was performed for pregnant women and blood donors separately. Marital status was not included in the blood donor modeling due to missing data. Cramer's V was used to assess the correlation between facilities and location of residence. Due to the moderate correlation of these two variables, location of residence (urban/suburban/rural) was chosen as the covariate for the logistic regression models instead of the facility.

### 2.4. Ethics

A waiver of consent was obtained from the CBCHS in-country institutional review board and an exemption was granted by the Institutional Review Board at the University of Alabama at Birmingham.

## 3. Results

At four CBCHS facilities in Cameroon, 7069 pregnant women and 4225 blood donors were screened for infection in 2014. Demographic data were available for 2827 ANC clients and 3364 blood donors from three of the four sites (Table 1). For pregnant women, the median age was 26 years and this did not differ across facilities (range 26-27 years; *p* = 0.177) whereas the residency location (urban/rural) differed significantly (*p* < 0.001). Women screened in Mutengene lived predominantly in urban settings while those in Banso were mostly from suburban and rural locales. Blood donors were significantly older than pregnant women (median age 33) and their median age differed across facilities (range 30–34 years; *p* value < 0.0001). The majority of blood donors were male (77.5%) and married (61.3%), had blood type O (63.4%), and had a positive Rh status (96.8%). These characteristics differed across sites.

The seroprevalence and 95% CI of the infections in pregnant women and donors by facility are shown in Table 2. Overall, the rates in pregnant women were 4.4% for hepatitis B, 6% for HIV, and 1.7% for syphilis. Among blood donors, seroprevalence was 6.8% for hepatitis B, 2.2% for HIV, and 4% for syphilis. Additional testing among blood donors showed that 1.7% had antibodies for hepatitis C and 1.9% had malaria. Furthermore, the prevalence rates in pregnant women varied significantly across facilities ranging from 1.1 to 9.6% for hepatitis B, from 3 to 10.2% for HIV, and from 1.3 to 3.8% for syphilis. The range among blood donors was 5.0–8.8% for hepatitis B, 1.4–2.8% for HIV, 3.3–4.5% for syphilis, and 0.5–2.5% for HCV. Pregnant women seen in Mutengene and Mboppi had a higher prevalence of HBV and HIV compared to women seen at Mbingo and Banso facilities. Blood donors did not show a consistent pattern of uniformly high or low prevalence at specific facilities.

Coinfection with more than one pathogen was detected in 2.9% of the pregnant women in Mutengene (13 HIV/HBV, 6 HIV/syphilis, 3 HBV/syphilis, and 3 HIV/HBV/syphilis). Rates of coinfection at Banso and Mbingo were very low at 0.3 and 0.2%, respectively. Among blood donors at the Mutengene facility, 1.8% had coinfection (8 HBV/syphilis, 7 HIV/HBV, and 1 HIV/syphilis) and the coinfection rate was 1% at Mbingo and 0.7% at Banso. Coinfection rates were not available from Mboppi.

Table 3 shows crude and adjusted odds ratios and 95% confidence intervals resulting from logistic regression analyses of hepatitis B, HIV, and syphilis infection as outcome variables among pregnant women. Pregnant women from urban locations were more likely to have hepatitis B (aOR 2.9, CI 1.6–5.4) and HIV (aOR 3.5, CI 1.9–6.7), compared to women from rural areas. Older age (aOR 1.04, CI 1.01–1.07) was also significantly associated with HIV status. The unadjusted model for blood donors showed lower rates of HIV in older donors and lower rates of hepatitis C infection for donors from suburban areas. Older age among blood donors was the only factor significantly associated with risk of syphilis in the adjusted model (aOR 1.02, CI 1.002–1.05) (Table 4).

## 4. Discussion

Infections in pregnancy remain common in Cameroon with 2014 seroprevalence rates as high as 9.6% for hepatitis B, 10.2% for HIV, and 3.8% for syphilis. Fortunately, vertical transmission of HBV, HIV, and syphilis can be prevented with currently available tools if the infection is detected in time. As evidence of how challenging it is to reduce infection rates, rates of HBV and HIV infection in pregnancy were also elevated 20 years ago (HBV 5.4%, HIV 3.5%) [21]. Rates of syphilis among pregnant women in Cameroon have improved from 15.8% to 4% over the past 20 years with increased access to syphilis screening and treatment programs [21–23]. Infection with hepatitis C is less common with a seroprevalence among blood donors in this study that matches a recent HCV seroprevalence study in health care workers (1.7%) [24]. Fortunately, this is significantly lower than the seroprevalence of 56% noted in an older Cameroon cohort (>60 years old). The HCV prevalence in the older group has been attributed to exposure to unclean needles during medical interventions in the early 20th century [25]. Very low rates of IV drug abuse in Cameroon and the infrequency of heterosexual transmission of HCV likely contributed to this cohort effect with a much lower prevalence noted in younger age groups [26, 27].

Our findings also show wide variability in infection rates depending on the facility site in Cameroon, particularly for hepatitis B (1.1–9.6%) and HIV (3.0–10.2%). This highlights the presence of subpopulations of pregnant women who warrant targeted testing and prevention efforts. Rates of infection were elevated at the sites which capture a higher population of urban pregnant women. Douala is the largest city in Cameroon and pregnant women seen at Mboppi Hospital in Douala had the second highest rates of HBV (4.3%) and HIV (5.9%). This finding is consistent with other studies, although there may be confounding explanatory factors (such as number of sexual partners) which were not captured in this analysis. Pregnant women seen in Mutengene also had significantly higher rates of HBV, HIV, and syphilis in the multivariate analysis and the highest rates of coinfection, particularly for HBV and HIV. Mutengene is a semiurban town with a population of 40,000 which is located at the junction of two major roadways. Many of the patients seen at the facility are itinerant or travel from long distances to receive medical care. Infection rates among blood donors at Mutengene were comparable to the infection rates seen in pregnant women, although HIV prevalence was lower (2.7%). Since most blood donors were male, the lower HIV prevalence noted in blood donors compared to pregnant women mirrors national HIV epidemiology in Cameroon where the prevalence of infection in males (2.9%) is about half of the rate documented among females (5.6%) [28].

Most of sub-Saharan Africa is endemic for HBV infection (defined as HBsAg + >8%). Antenatal HBV screening has become routine practice at the larger CBCHS facilities but is not standard practice nationwide. The importance of screening for hepatitis B in pregnancy is highlighted by the fact that vertical transmission rates are 70–90% in women with chronic hepatitis B and a positive hepatitis B e antigen (HBeAg) and 10–40% if the HBeAg is negative. Chronic HBV infection develops in 90% of infected neonates, compared to 5–10% of infected immunocompetent adults. Vertical transmission of hepatitis B is preventable in 95% of cases if the exposed infant receives prophylaxis with birth dose univalent HBV vaccine followed by completion of the series. The birth dose vaccine is not part of the national vaccine plan in Cameroon or in many other countries in sub-Saharan Africa which instead prioritize HBV-containing pentavalent vaccine starting at 6 weeks of age. The pentavalent vaccine may not prevent vertical transmission of HBV. If vertical transmission is common, addition of the univalent HBV birth dose vaccine to the current vaccine program would be one effective way to reduce pediatric HBV infection rates [29, 30]. Neonatal BCG vaccine is administered routinely in most sub-Saharan countries and univalent HPV vaccine can be administered at the same time as soon as possible after birth.

The prevalence of HIV at CBCHS antenatal clinics in Cameroon has steadily decreased from 10.5% in 2004 to 3.4% in 2014 [31]. The 2011 Cameroon Demographic and Health Survey showed that women of childbearing age in the two largest urban cities had an HIV prevalence of 7.7%, compared to 4.6% among women from rural areas, highlighting the significance of regional variation [32]. Our findings support these trends but underscore the need for sustained efforts in high risk areas despite encouraging national trends [32]. The reduction in HIV prevalence in Cameroon has occurred in the setting of a robust Prevention of Mother to Child HIV Transmission (PMTCT) program with funding from the Elizabeth Glaser Pediatric AIDS Foundation and the US President's Emergency Plan for AIDS Relief (PEPFAR).

WHO launched a global initiative in 2007 to eliminate congenital syphilis. They recommended screening for syphilis at the initial antenatal clinic visit with a goal of 95% and a follow-up screening test during the third trimester [33]. Despite these efforts, syphilis rates are increasing, antenatal screening is not universal, and adverse infection outcomes occur in an estimated 206,000 pregnancies each year in sub-Saharan Africa [34]. These outcomes include stillbirth, neonatal death, low birth weight, and congenital syphilis. Fortunately, treatment with penicillin remains highly effective, affordable, and well tolerated. Our findings suggest the importance of screening for syphilis.

In this study, infections with bloodborne pathogens were commonly documented among routine voluntary blood donors. These rates are aligned with prior studies; at one hospital in urban Cameroon, nearly 15% of the donated blood screened positive for infections with HIV, HBV, HCV, or* T. pallidum* [35, 36]. Transfusion in this setting poses a real risk of infection for women who require blood products during pregnancy or in the postpartum setting and 276 pregnant women in Cameroon received blood transfusions at one of the four facilities in 2014. Another concern is the potential for transmission of bloodborne pathogens associated with fetal risk for which testing is not performed (i.e., CMV or toxoplasmosis). Although routine screening for malaria in donated blood products was only performed at one of the four sites, this should be routinely performed since malaria is endemic in Cameroon and pregnant women are more susceptible to this infection and its consequences [37, 38].

Another issue common to sub-Saharan Africa is the source of the blood products used for transfusion. One large study of blood donors in Cameroon showed that most donors (64%) were family members of the patient [39]. One of the four key components of the WHO blood safety plan is to improve donor recruitment and collection by restricting collection to products from low-risk donors. This specifically excludes friends and family members. The ideal donors are voluntary, nonremunerated, and recruited through a centralized system, such as blood centers which are independent from hospital facilities [12]. Major challenges persist related to the safety and expense of blood product transfusion in sub-Saharan Africa and the current supply only meets 40% of the estimated need [40].

The strengths of our study include the size of the cohort and the ability to compare contemporaneous ANC seroprevalence data with voluntary blood donors at four large clinical sites. In terms of study limitations, only certain demographic information was available for pregnant women and individual-level data was not available for one of the testing sites. Also, although the characteristics of the tests used were excellent, viral load testing for HBV or HIV, nontreponemal testing for syphilis to identify active infection, and microscopy to confirm malaria infection were not performed. This may have led to an overestimation of syphilis infection rates and an underestimation of malaria infection rates.

## 5. Conclusions

Antenatal care provides an excellent opportunity to screen women for infections which are common and treatable and can be transmitted vertically. Hepatitis B, HIV, and syphilis infections remain common in pregnant women and blood donors in Cameroon and the prevalence rates vary by site. Timely access to quality testing and treatment can prevent vertical transmission, but ongoing attention to blood safety is also needed. Policy changes at the national and international levels would go further to ensure that early access to antenatal testing and treatment or prevention is widely available in sub-Saharan Africa where disease prevalence is high.

## Figures and Tables

**(a):** 

Pregnant women	Mbingo *n* = 1230	Mutengene *n* = 871	Banso *n* = 726	Total *n* = 2827	*p* value^*∗∗*^
*Age, median (range)*	26 (14–55)	27 (15–44)	26 (14–53)	26 (4–55)	0.177

*Residence *					
Urban	123 (35.0)	735 (84.4)	56 (7.7)	914 (46.9)	<0.0001
Suburban	87 (24.8)	136 (15.6)	398 (54.8)	621 (31.9)	
Rural	141 (40.2)	—	272 (37.5)	413 (21.2)	

**(b):** 

Blood donors	*n* = 1305	*n* = 889	*n* = 1170	*n* = 3364	*p* value^*∗∗*^
*Age, median (range)*	30 (15–75)	34 (17–56)	32 (17–63)	33 (15–75)	<0.0001

*Gender*					
Male	920 (70.6)	807 (90.8)	878 (75.2)	2605 (77.5)	<0.0001
Female	384 (29.4)	82 (9.2)	290 (24.8)	756 (22.5)	

*Residence (n* = 2999)					
Urban	480 (51)	596 (67)	61 (5.2)	1138 (38.0)	<0.0001
Suburban	116 (12.3)	293 (33)	839 (71.8)	1248 (41.6)	
Rural	345 (36.7)	—	268 (23)	613 (20.4)	

*Marital status (n* = 2069)					
Married	69 (56)	507 (57)	693 (65.6)	1269 (61.3)	0.0002
Single^*∗*^	55 (44)	382 (43)	363 (34.4)	800 (38.7)	

*Blood type*					
A	226 (17.4)	109 (12.3)	243 (20.8)	578 (17.2)	<0.0001
B	282 (21.7)	82 (9.2)	211 (18.1)	575 (17.1)	
AB	27 (2.1)	7 (0.8)	42 (3.6)	76 (2.3)	
O	767 (58.9)	689 (77.7)	672 (57.5)	2128 (63.4)	

*Rh status*					
Positive	1251 (95.9)	875 (98.4)	1129 (96.6)	3255 (96.8)	0.0033
Negative	54 (4.1)	14 (1.6)	40 (3.4)	108 (3.2)	

^*∗*^Demographics were available at 3 of the 4 study sites.

^*∗∗*^ *p* value is to test the overall difference in seroprevalence for each categorical variable (*χ* ^2^ test) or the median difference of the continuous variable.

**(a):** 

Pregnant women	Mboppi (*n* = 4242)	Mbingo (*n* = 1230)	Mutengene (*n* = 871)	Banso (*n* = 726)	*p* value	Total (*n* = 7069)
Hepatitis B	184 (4.3) (3.7, 5.0)	14 (1.1) (0.6, 1.9)	84 (9.6) (7.7, 11.8)	26 (3.6) (2.3, 5.2)	<0.0001	308 (4.4) (3.9, 4.9)
HIV	252 (5.9) (5.3, 6.7)	56 (4.6) (3.5, 5.9)	89 (10.2) (8.3, 12.4)	22 (3.0) (1.9, 4.6)	<0.0001	413 (6.0) (5.3, 6.4)

Syphilis	57 (1.3) (1.0, 1.7)	17 (1.4) (0.8, 2.2)	33 (3.8) (2.6, 5.3)	15 (2.1) (1.2, 3.4)	<0.0001	122 (1.7) (1.4, 2.1)

**(b):** 

Blood donors	(*n* = 861)	(*n* = 1305)	(*n* = 889)	(*n* = 1170)	*p* value	(*n* = 4225)
Hepatitis B	50 (5.8) (4.3, 4.6)	115 (8.8) (7.3, 10.5)	64 (7.2) (5.6, 9.1)	58 (5.0) (3.8, 6.4)	0.001	287 (6.8) (6.1, 7.6)

HIV	24 (2.8) (1.8, 4.1)	30 (2.3) (1.6, 3.3)	24 (2.7) (1.7, 4.0)	16 (1.4) (0.7, 2.2)	0.11	94 (2.2) (1.8, 2.7)

Syphilis	39 (4.5) (3.2, 6.1)	43 (3.3) (1.7, 3.4)	40 (4.5) (3.2, 6.1)	46 (3.9) (2.9, 5.2)	0.40	168 (4.0) (3.4, 4.6)

Hepatitis C	20 (2.3) (1.4, 3.6)	32 (2.5) (2.4, 4.4)	4 (0.5) (0.01, 1.2)	15 (1.3) (0.7, 2.1)	0.001	71 (1.7) (1.3, 2.1)

Malaria	N/A	N/A	N/A	22 (1.9)	N/A	22/1170 (1.9) (1.2, 2.8)

^*∗*^Infections are not mutually exclusive; coinfection data is discussed in Section 3.

**Table 3:** Predictors of hepatitis B, HIV, and syphilis infection in pregnant women.

Variable	Hepatitis B infection		HIV infection		Syphilis infection	
	Unadjusted OR (95% CI)	Adjusted OR (95% CI)	Unadjusted OR (95% CI)	Adjusted OR (95% CI)	Unadjusted OR (95% CI)	Adjusted OR (95% CI)
		*n* = 1904		*n* = 1904		*n* = 1904

Age	(*n* = 2758)		(*n* = 2758)		(*n* = 2758)	
Continuous	1.00 (0.98, 1.03)	1.00 (0.97, 1.04)	1.03 (1.01, 1.06)^*∗*^	1.04 (1.01, 1.07)^*∗*^	1.00 (0.96, 1.04)	1.01 (0.96, 1.05)

Location	(*n* = 1948)		(*n* = 1948)		(*n* = 1948)	
Urban	3.1 (1.7, 5.7)^*∗*^	2.9 (1.6, 5.4)^*∗*^	3.7 (2.0, 7.0)^*∗*^	3.5 (1.9, 6.7)^*∗*^	1.5 (0.7, 3.2)	1.6 (0.7, 3.6)
Suburban	1.3 (0.7, 2.7)	1.3 (0.7, 2.7)	2.1 (1.1, 4.2)^*∗*^	2.1 (1.1, 4.3)^*∗*^	1.0 (0.4, 2.3)	1.1 (0.4, 2.6)
Rural	REF	REF	REF	REF	REF	REF

^*∗*^Indicating statistical significance. REF: reference.

**Table 4:** Predictors of hepatitis B, C, HIV, and syphilis in blood donors.

Variable	Hepatitis B infection		Hepatitis C infection		HIV infection		Syphilis infection	
	Unadjusted OR (95% CI)	Adjusted OR (95% CI)	Unadjusted OR (95% CI)	Adjusted OR (95% CI)	Unadjusted OR (95% CI)	Adjusted OR (95% CI)	Unadjusted OR (95% CI)	Adjusted OR (95% CI)
		(*n* = 2988)		(*n* = 2988)		(*n* = 2986)		(*n* = 2987)

Age	(*n* = 3352)		(*n* = 3352)		(*n* = 3351)		(*n* = 3351)	
Continuous	1.00 (0.98, 1.01)	1.0 (0.98, 1.02)	1.03 (1.0, 1.06)	1.00 (0.98, 1.02)	0.96 (0.94, 0.99)^*∗*^	0.97 (0.94, 1.005)	1.03 (1.01, 1.05)^*∗*^	1.02 (1.002, 1.05)^*∗*^

Location	(*n* = 2999)				(*n* = 2997)		(*n* = 2998)	
Urban	1.4 (1.0, 2.1)	1.4 (0.9, 2.1)	0.6 (0.3, 1.4)	1.4 (0.9, 2.1)	1.4 (0.6, 2.8)	1.3 (0.6, 2.8)	1.2 (0.7, 2.1)	1.2 (0.7, 2.0)
Suburban	1.0 (0.7, 1.5)	1.0 (0.6, 1.4)	0.4 (0.2, 0.9)^*∗*^	1.2 (0.8, 1.9)	1.1 (0.5, 2.3)	1.1 (0.5, 2.3)	1.3 (0.8, 2.2)	1.3 (0.7, 2.2)
Rural	REF	REF	REF	REF	REF	REF	REF	REF

Gender	(*n* = 3361)		(*n* = 3361)		(*n* = 3359)		(*n* = 3360)	
M	REF	REF	REF	REF	REF	REF	REF	REF
F	1.0 (0.7, 1.3)	0.9 (0.6, 1.3)	1.1 (0.6, 2.0)	0.8 (0.6, 1.2)	0.6 (0.3, 1.2)	0.5 (0.2, 1.2)	0.8 (0.5, 1.2)	0.8 (0.5, 1.3)

^*∗*^Indicating statistical significance. REF: reference.
